# Effect of Vitamin D Supplementation on Procalcitonin as Prognostic Biomarker in Patients with Ventilator Associated Pneumonia Complicated with Vitamin D Deficiency

**Published:** 2017

**Authors:** Amir Ebrahim Miroliaee, Jamshid Salamzadeh, Shervin Shokouhi, Alireza Fatemi, Seyed Hossein Ardehali, Mohammad Reza Hajiesmaeili, Zahra Sahraei

**Affiliations:** a *Clinical Pharmacy Department, School of Pharmacy, Shahid Beheshti University of Medical Sciences, Tehran, Iran. *; b *Department of Infectious Diseases, Loghman Hospital, Shahid Beheshti University of Medical Sciences, Tehran, Iran. *; c *Infectious Diseases and Tropical Medicine Research Center, Shahid Beheshti University of Medical Sciences, Tehran, Iran. *; d *Department of Critical Care, Shohadaye-Tajrish Hospital, Shahid Beheshti University of Medical Sciences, Tehran, Iran. *; e *Loghman Clinical Research Development Center, Shahid Beheshti University of Medical Sciences, Tehran, Iran.*

**Keywords:** Procalcitonin, VAP, CPIS score, SOFA score, Vitamin D

## Abstract

Ventilator-associated pneumonia (VAP) is a common and serious problem that develops after more than 48 h of mechanical ventilation. Improving the activity of immune system with vitamin D, and its consequent impact on prognostic biomarkers of VAP was studied in the current study.

A randomized double blind placebo controlled clinical trial was designed. A total of 46 patients with VAP, who were suffering from vitamin D deficiency, were randomly allocated into the study groups of placebo (n=22) and treatment (n=24) The treatment group received 300,000 units of intramuscular vitamin D. Serum levels of procalcitonin and vitamin D along with SOFA and CPIS scores were determined at baseline and on day 7 after intervention. The mortality rate of patients was also monitored for the succeeding 28 days after the injection.

The administration of vitamin D significantly enhanced its levels (P<0.0001) in the treated patients (12.28 ± 8.26) in comparison to placebo group (1.15 ± 1.50). The levels of PCT were significantly decreased (p=0.001) in the treatment group (– 0.02 ± 0.59 ng/mL) compared to that of placebo group (0.68 ± 1.03 ng/mL). However, changes in (SOFA) and CPIS scores were not significantly different between study groups (p=0.63 and p=0.32, respectively). Interestingly, the mortality rate of patients in the treatment group (5/24) was significantly lower (p=0.04) than that of the placebo group (11/22).

In conclusion, our results indicate that vitamin D supplementation can significantly reduce the procalcitonin in (VAP) patients, and must be considered as a preventive and/or therapeutic strategy.

## Introduction

Ventilator associated pneumonia (VAP) is a common and serious hospital-acquired infection developing after more than 48 h of mechanical ventilation. The estimated incidence for this condition is 10-25%, while the all-cause mortality reaches 25-50% (1, 2). (VAP) is generally manifested by a progressive pulmonary infiltration and patients usually suffer from various symptoms such as fever, purulent discharge from trachea, leukocytosis, tachypnea , and reduced oxygenation (3).

Human airways are usually exposed to microbes and pollutants. This exposure is usually limited to the upper respiratory tract, and only during micro-aspiration, pathogenic microorganisms can enter the lower respiratory tract. Subsequently, the innate and adaptive immune system deals with the intruder microorganisms and eliminates or prevents from potential infections. However, in the case of immunity dysfunction, infections occur. Thus, in patients with weakened immune system, boosting the immune system must be considered as a preventive or therapeutic strategy (4, 5).

Supplementation of vitamin D has been confirmed to improve the immune status. Vitamin D receptors (VDRs) have been found on different immune cells such as neutrophils, macrophages, and dendritic cells. Vitamin D not only activates the T lymphocytes, but also reduces the secretion of pro-inflammatory cytokines such as TNF-α, interleukin-1 (IL-1), IL-6, IL-8 and IL-12 , resulting in improved function of the immune system and prevention of damages associated with hyper activation of inflammatory processes. Therefore, vitamin D is believed to have immune modulatory effects (6). Recently some other effects like neuroprotection by modulation of endothelial progenitor cells and (NFκB) have been reported (7). 

In the recent years, it has been reported that vitamin D deficiency is prevalent in both genders in Iran, reaching 85% of general population in Tehran (8, 9). This has been associated with the clothing habits in the society and a major lack of sea food in Iranian diet as well as air pollution. Furthermore, other studies have shown that vitamin D deficiency negatively affects the prognosis of patients with community acquired pneumonia. Moreover, it has been demonstrated that the treatment of vitamin D deficiency in children reduces the recurrence of pneumonia (10-12).

Procalcitonin has been established as an infection marker due to its level increases in bacterial infections. In (VAP), macrophage alveoli that have phagocytized microorganism will release pro-inflammation cytokine such as TNF-α and IL-1β that will stimulate macrophage cells and lung neuroendocrine to produce (PCT). Sequential organ failure assessment (SOFA) score and clinical pulmonary infection score (CPIS) are among markers that have been suggested in the recent years for prediction of prognosis and outcome in patients with pneumonia. These three indices have also been confirmed to be useful in prediction of outcome for patients with hospital-acquired pneumonia.As.such, ssessment of patients for these indices can indicate appropriateness of treatments. Decrease in these scores is related to improvements in the health status of patients (13, 14). It has been shown that increase in PCT levels is associated with poor outcomes. In addition, its decline from day 3 to day 7 of diagnosis of (VAP) could act as a predictor for treatment response (15, 16). In patients with Community acquired pneumonia (CAP), treatment decisions aided by PCT guidance could result in a lower antibiotic use, hospital admission, and length of hospital stay (17). Similarly, the use of this biomarker can also reduce antibiotic use in patients with VAP (18). Procalcitonin is an inflammatory marker which is induced by pro-inflammatory cytokines Furthermore, vitamin D can result in a reduction of the pro-inflammatory cytokines and thus, is supposed to indirectly reduce procalcitonin levels in serum (6).

According to a report by Tellioglu *et al.*, administration of a single intramuscular mega dose (600,000 units) of vitamin D in elderly patients could improve vitamin D levels in a linear pattern, so that 100% of patients were maintaining optimal vitamin D levels at 12^th^ week (19). This mega dose was also well tolerated and safe in the study population (19). Therefore, being judicious, we decided to use an intramuscular injection of a 300,000 units of vitamin D in the current study.

This study aims to investigate the effects of vitamin D administration on disease outcomes, and procalcitonin that plays as prognostic biomarker associated with mortality of patients with VAP.

## Experimental

*Study design and participants*

A randomized double blind placebo controlled clinical trial was run in two university hospitals, Loghman-e-Hakim and Shohadaye Tajrish in Tehran, Iran. The subjects were >18 years old who had been diagnosed with VAP based on the following diagnostic criteria: The enrolled subjects showed new or progressive infiltrations in chest radiography within 48-72 h after mechanical ventilation and also had at least two of the three following criteria: 1) fever >38 °C, 2) leukocytosis or leukopenia (WBC<4000/mcl or WBC>12000/mcl) 3) purulent respiratory secretions.

Subjects with chronic renal failure (GFR <30cc/min), pancreatitis, hepatic insufficiency with stage B or C of child-Pugh score, those with a history of cancer in the last 3 month or subjects undergoing chemotherapy, immune compromised patients, patients with VAP but with normal vitamin D levels (≥ 30 ng/mL) and those with coagulopathy (INR>1.5 or PTT>2 times of normal range or plt<100000/mL) were excluded from the study. Furthermore, a written informed consent was obtained from the patients or their concerned family members. The study was approved by the ethics committee of Shahid Beheshti University of Medical Sciences, Tehran, Iran 

This study was registered with (IRCT.IR) Iranian Registry of Clinical Trials, number IRCT2014112920134N1.

*Sample size estimation and randomization*

Estimated sample size for each group was 23 patients, with α=0.05 and power=80%. Considering a 20% dropout rate, the total sample volume was raised to 28 in each group.

The "RAND" command of the Microsoft Excel^®^ 2013, was used for randomized allocation of the patients in the two equal sized study groups (n=23). 

*Procedure*

Eligible patients were randomly allocated into two study groups of treatment and placebo within 48 h after diagnosis of (VAP). Treatment group received intramuscular vitamin D, 300,000 units, and the placebo group received the same dosage form containing exact composition of drug formulation except vitamin D as the main ingredient. Before inclusion of the patients in the study and after checking for other inclusion and exclusion criteria, their serum level of 25-hydroxy vitamin D was determined. Those with vitamin D deficiency underwent procalcitonin level measurements and their medical and medication history along with (SOFA) score, CPIS score, Acute Physiology and Chronic Health Evaluation II (APACHE II) score, renal and hepatic function, the status of respiratory secretions, pneumonia-related clinical findings, complete blood count (CBC), temperature and vital signs were obtained and recorded before allocation into the study groups. Vitamin D level in subjects were divided into 4 categories: <10 ng/mL = severely deficient, 10-20 ng/mL = deficient, 20-30 ng/mL = insufficient, and >30 ng/mL as normal. 

On the seventh day after intervention, serum 25-hydroxy vitamin D and procalcitonin levels as well as (SOFA) score and (CPIS) score were again determined. Pneumonia-related clinical findings also were checked.

On the first day based on empirical diagnosis, the antibiotic therapy of patients was done according to the Infectious Diseases Society of America (IDSA) guidelines. This was applied in both hospital settings. The therapeutic regiment for all patients included vancomycin plus carbapenem and ciprofloxacin or aminoglycosides. In the case of lack of response to empirical therapy or based on the culture results, the treatment protocol was modified. 

*Primary and secondary outcomes*

At the end of the study, the effect of vitamin D administration on the studied markers procalcitonin, (SOFA) score and (CPIS) score were evaluated as the primary outcomes. The morality rate among study subjecs were also recorded after 28 days as a secondary outcome. 

*Statistical analysis*

The statistical analyses with appropriate parametric, *i.e.* the student’s t-test, and non-parametric, *i.e.* the Mann-Whitney U test, for quantitative data, and the Chi-square test or Fisher exact test for qualitative data, were performed using Statistical Package for Social Sciences (SPSS) version 18.0.

## Results

**Figure 1:**
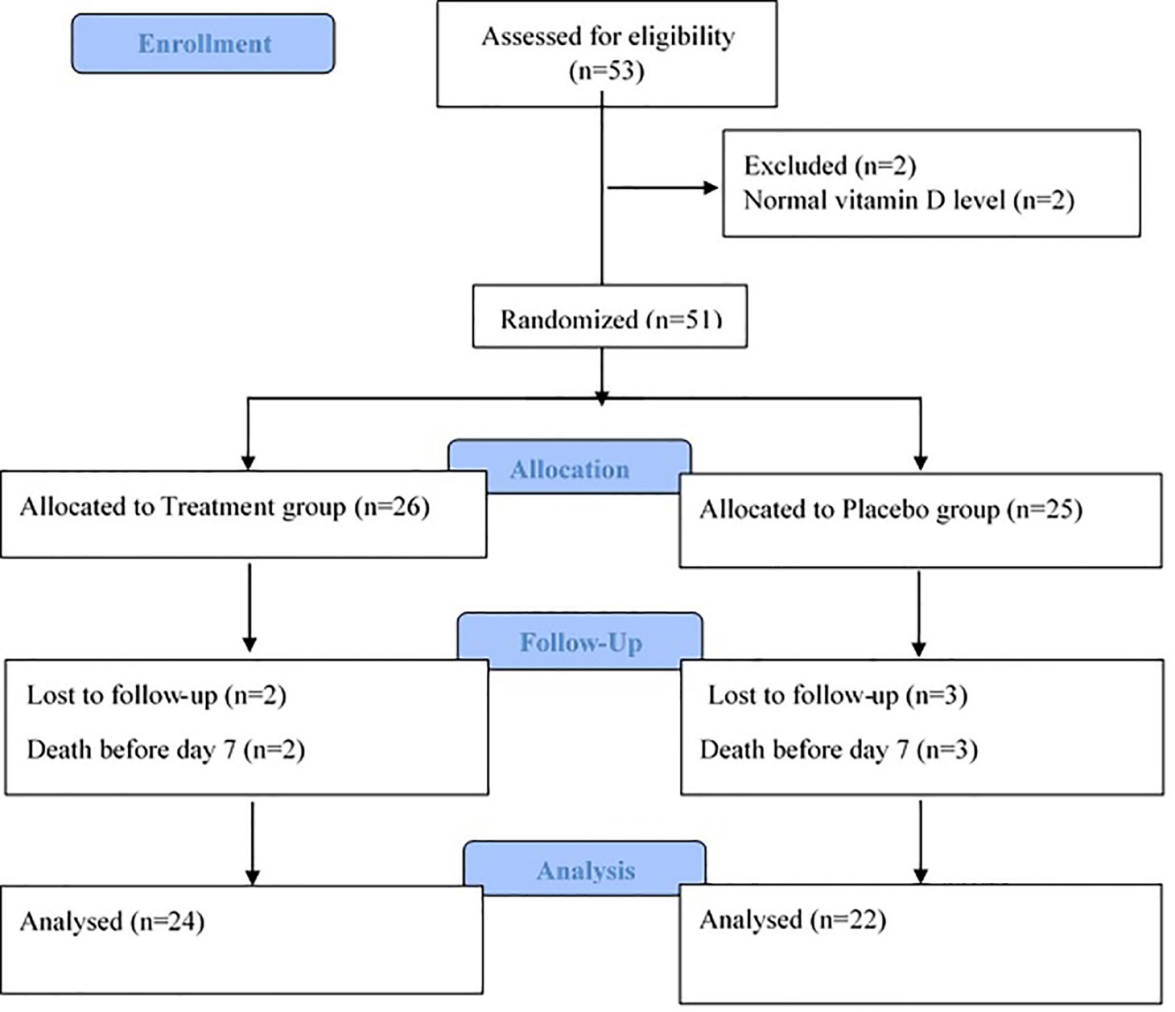
Flow chart of the trial

Fifty three (VAP) patients with desirable criteria were evaluated from October 2014 till July 2015 Tow subjects were excluded from the study due to normal vitamin D levels. From 51 patients enrolled in the study, 5 patients died before day 7^th^, and as such, 46 patients including 29 males and 17 females could complete the study, 22 in placebo and 24 in the treatment groups. Flow diagram of the study is presented in the Figure 1.

**Figure 2:**
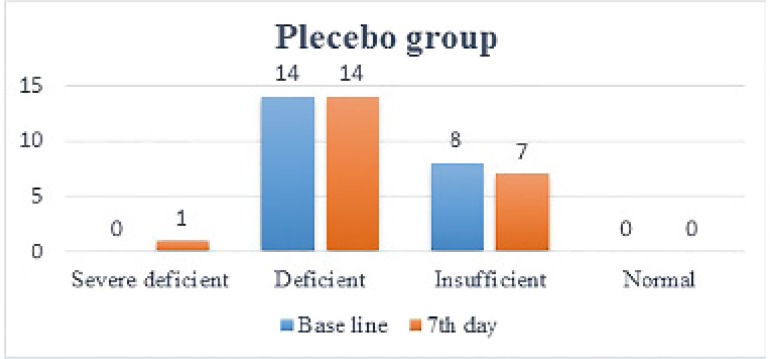
Numbers of patients in each vitamin D level in placebo group at baseline and at 7^th^ day of treatment.

**Figure 3:**
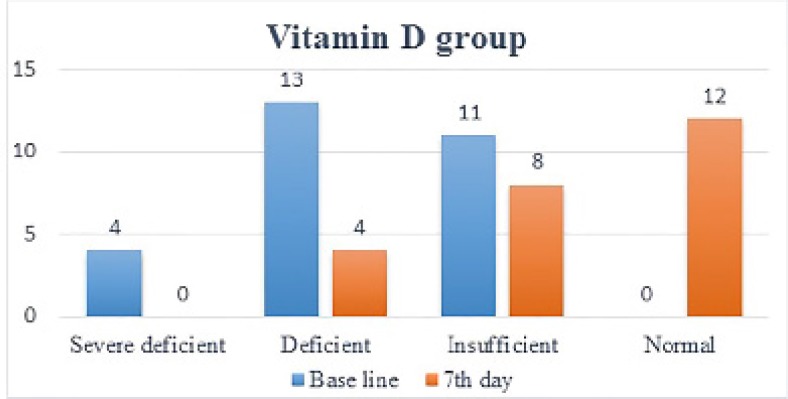
Numbers of patients in each vitamin D level in treatment group at baseline and at 7^th^ day of treatment

**Figure 4:**
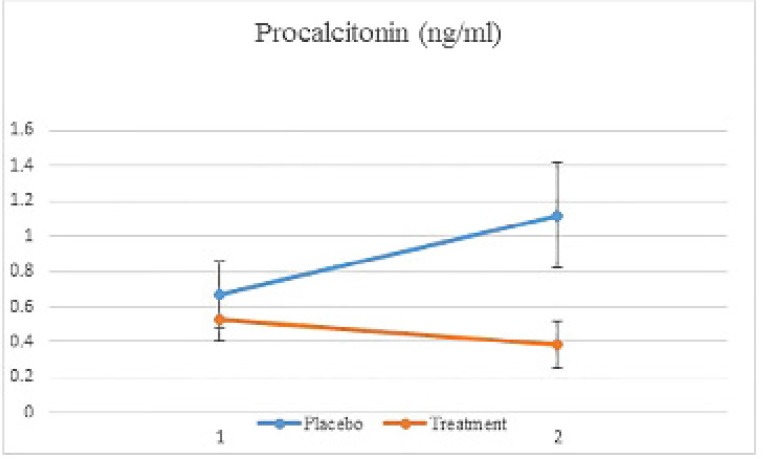
Procalcitonin plasma level from baseline till the 7^th^ day after vitamin D administration. (Mean ±SE

**Table 1:** The demographic and baseline study variables in the study groups

**Parameter**	**Placebo group (Mean ±SD)**	**Treatment group(Mean ±SD)**	***P*** **-value**	**95** ** % Confidence interval**
Age (year)	56.45 ± 20.70	57.83 ± 18.84	0.68	-
Gender	13 (M), 9 (F)	16 (M), 8 (F)	0.6	-
Weight (Kg)	70.73 ± 7.66	74.58 ± 11.01	0.18	[–9.54, 1.83]
Vitamin D (ng/mL)	19.5 ± 4.60	17.12± 6.11	0.15	[–0.86, 5.61]
SOFA score	7 ± 1.70	7 ± 1.02	0.63	-
CPIS score	7.64 ± 0.79	7.83 ± 0.76	0.32	-
PCT (ng/ mL)	0.67 ± 0.88	0.53 ± 0.61	0.91	-
APACHE II score	21.45 ± 5.69	25.12 ± 4.49	0.02	[–6.70, -0.63]
WBC (count/ mL)	13153.5 ±5742.7	10357.1± 4484.5	0.8	[-5982.1, 387.4]
Temperature (°C)	37.77 ± 0.77	38.1 ± 0.75	0.81	[-.2, 0.74]

SD: Standard Deviation; M: Male; F: Female; Kg: Kilogram; ng: Nanogram; mL: Milliliter; WBC: White blood cell; ^°^C: Degree Celsius.

**Table 2:** Procalcitonin levels in the treatment and placebo groups at baseline and at the 7^th^ day after vitamin D administration

**Parameter **	**Placebo group (mean ± SD) **	**Treatment group (mean ± SD)**	**Significance**
Procalcitonin baseline (ng/mL)	0.67 ± 0.88	0.53 ± 0.61 ng/mL	P=0.91
Procalcitonin 7th day (ng/mL)	1.12 ± 1.40	0.39 ± 0.63	P= 0.001
Procalcitonin difference (ng/mL)	0.68 ± 1.03	– 0.02 ± 0.59	P=0.001

**Table 3:** Differences of study outcomes between study groups at baseline and the 7^th^ day after vitamin D administration.** (**Mean ± SD

**Parameter**	**Treatment group**	**Placebo group**	**Significance**
SOFA score	0.58 ± 1.25	0.09 ± 2.60	P= 0.37 *
CPIS score	1.92 ± 1.53	1.5 ± 1.80	P= 0.46 *
WBC	3714.3 ± 3900	2077 ± 6582	P= 0.57 **
Temperature (°C)	.99 ± 1.1	.03 ± 1.3	P= 0.2 **

* Data analyzed by Mann-whitney

** or T-test (SOFA) Sequential organ failure assessment; CPIS: Clinical pulmonary infection score; WBC: White blood cell; °C: Degree Celsius.

**Table 4:** The dominant microorganisms causing infection in the study groups

**Organism **	**Treatment group (n)**	**Significance **	**Placebo group (n)**
*Acinetobacter baumannii*	8	0.4 *	10
*Klebsiella pneumoniae*	3	0.7 **	4
*Staphylococcus aureus*	5	0.4 **	2
*Enterobacter species*	2	1 **	2
*Pseudomonas Aeruginosa*	2	1 **	1
*Negative culture *	4	1 **	3

* Data analyzed by the Fisher’s exact test

** or Chi- square test

Among the severity score and mortality estimation tools used in this study, the baseline (APACHE) II score was significantly lower in the placebo group compared to treatment group (p=0.02). For the baseline (SOFA) and CPIS, as a surrogate tool to aid the diagnosis of VAP, differences were not statistically significant (p=0.63 and 0.32, respectively). Other baseline parameters did not show any significant difference between two groups. (Table 1)

One week after administration of intramuscular vitamin D, its serum levels in the treatment group increased dramatically (12.28 ± 8.26) (p<0.0001). In 19 patients, vitamin D levels increased by one level (9.11 ± 4.4), in 5 patients, it increased by two levels (24.3 ± 8.8), so that in 12 patients, vitamin D levels became normal. As mentioned in the method, vitamin D level has been categorized between severe deficiency and normal into 4 level. This is while in placebo group, no significant change (p=0.78) in vitamin D levels was noted (1.15 ± 1.50) and in 2 patients vitamin D levels dropped by 1 level (Figure 2 and 3).

In the treatment group, procalcitonin levels dropped significantly from the first (baseline) to 7^th^ day of study , while in the placebo group, its levels were shown to increase significantly (p=0.001). (Table 2 and Figure 4).

Changes in (SOFA) and (CPIS) scores between the treatment and placebo groups were not significant (p=0.37 and p=0.46, respectively). Although, (SOFA) score had a marginal improvement in the treatment group, the difference was not significantly different. Also there were a greater reduction in the fever and (WBC) count of the treatment group compared to those of the placebo group; however, no significant difference between two groups were obtained (Table 3).

Within 28 days after vitamin D administration, the mortality of patients in the treatment group (5/24) was significantly lower than that of the placebo group (11/22) (p=0.04). Since the mortality of patient is also dependent on the organism underlying the infection and therapeutic regiment, these parameters were also taken into consideration; however, no significant difference was found. The dominant microorganism in both groups was Acinetobacter followed by Klebsiella and multi-drug-resistant *Staphylococcus aureus* (MDRS). (Table 4)

The therapeutic regime was modified for one patient in the treatment group and for three patients in the placebo group. In the rest of the patients, after the culture results, de-escalation took place. The main modification in the therapeutic regime was the addition of colistin. 

## Discussion

In this randomized double blind placebo control study, we tried to assess the effect of vitamin D supplementation on prognostic marker and tools of mortality in (VAP) patients. The main hypothesis was reduction of prognostic biomarker of mortality in these patients by using of anti-inflammatory and immune modulatory effects of vitamin D.

The current study indicated that vitamin D deficiency is prevalent in the studied (VAP) patient population (96%) and that administration of a high dose vitamin D could restore its levels in patients significantly. The principal results of this study was that vitamin D administration could reduce procalcitonin levels in the serum of (VAP) patients, which was also correlated with a lower mortality rate in the intervention group versus placebo These observations were independent of the type of microorganism causing the infection Therefore, vitamin D supplementation can be considered as an appropriate therapeutic strategy in these patients. 

In previous studies focusing on vitamin D deficiency in healthy individuals in various cities in Iran, 75-85% of people were found to have vitamin D levels lower than normal (8, 9) They concluded that vitamin D deficiency can be caused by skin complexion, inadequate exposure to the sun, vegetarian diets, low consumption of sea food, lack of vitamin D food fortification program, and air pollution (8) Accordingly, the deficiency in chronic or hospitalized patients was already expected. Several approaches such as daily vitamin intake or administration of an annual high dose have been tried for replenishing vitamin D levels in individuals (19-22).

Ilahi *et al*. have shown that intramuscular administration of a 100,000 unit vitamin D, can increase chole calciferol levels to its peak levels within 1 week (23). In recent trials even one injection of 600,000 units of vitamin d, for rapid correction of its deficiency have been used effectively, without any significant adverse event in Schizophrenic patients (24). In the current study, similar results were obtained confirming that vitamin D levels can be restored within one week after its administration.

Recent studies have also shown that procalcitonin is a prognostic biomarker in patients with community acquired pneumonia (CAP) It has been shown that the decreasing trend of this marker in days 3-7 of the treatment can guide the antimicrobial regimen from parenteral to oral regimen, or decision making on discharging patient from hospital. When this marker is used along with other laboratory parameters such as (CRP) and (WBC) patient outcome can be predicted more precisely (17).Procalcitonin levels is now considered as a marker for determining pneumonia severity as well as the antibiotic choice and hospitalization decisions in patients with (CAP) (13, 15, 18). Procalcitonin levels are usually increased in bacterial infections, but not in viral infections. This biomarker can thus be potentially used to reduce the use of antibiotics in non-bacterial infections (18). It is also routinely used for determining the therapeutic protocol for patients with sepsis. Currently, procalcitonin evaluation has been included in guidelines for treatment of patients with sepsis (25).

Several studies have focused on evaluating PCT as a biomarker in assessment of health status of patients with (VAP) and the treatment outcome. Cleophas *et al*. have shown that if PCT is > 5 ng/mL in day 3 and > 0.5 ng/mL in day 7 of the treatment, it can be used to predict bad prognosis for VAP patients with 88.5% sensitivity and 53.2 Specificity on day 3 and 96.3% sensitivity and 66.7% Specificity on day 7 (16). In another study by Seligman *et al*. the reduction of PCT to < 0.47 ng/mL on day 4 of the treatment has been associated with reduced mortality of patients. In this study, among other markers such as CRP, copeptin and MR-PROANP, PCT was recognized as the most precise marker for predicting the mortality of patients (13). In another study on prognostic markers in patients with (VAP), procalcitonin reduction was significantly associated with reduced mortality rate in patients, so that a significant reduction in procalcitonin levels from day 4 of the treatment was related to survival with an odds ratio of 4.43 (95% confidence interval: 1.08 to 18.18) (26).

In the current study, (PCT) levels reached below 0.5 ng/mL on day 7 of the treatment, which can be a representative of improved outcome in treated patients. However, in the placebo group, procalcitonin levels not only did not drop, but also showed an increasing trend with an average of two-fold higher than the cut points reported by the previous studies (16). which is well in line with the higher rate of mortality in this group. 

Khoo *et al*. in a review article has cited that vitamin D receptors are present in different immune cells such as dendritic cells, monocytes and neutrophils. Interaction of vitamin D with its receptors can lead to a reduction in secretion of inflammatory cytokines such as IL-1, IL-6, IL-8, IL-12 and TNF-a herefore, vitamin D can indirectly decrease procalcitonin levels by reduction of these inflammatory mediators (6). The results of the current study confirmed the beneficial effects of vitamin D in reducing procalcitonin levels in (VAP) patients, which is in accordance with the mechanism of actions described above.

Yang *et al.* have shown that a reduction in (CPIS) score in patients with pneumonia can be an indicator applied to choose the appropriate antimicrobial treatment for the subjects and to predict their mortality rate (27). In a study by Luna *et al*. it was demonstrated that the reduction of CPIS score to below 6 in days 3-5 of the treatment is an indicator of reduced mortality in patients (28). In the current study, the reduction of this marker in both groups was similar, and in none of the treatment groups, (CPIS) score average was below 6 on day 7 of the treatment. Similarly, in a study by Seligman *et al*. no correlation between the reduction of CPIS score and patient mortality was noted (13).

Povoa *et al.* have reported that (SOFA) score on day 1 can predict mortality in patients with VAP. In this study, (SOFA) score was significantly lower in surviving patients (29). Although, no significant difference in (SOFA) score was found between the two patient groups, an improving trend was noted in the patients received vitamin D. Reasons for the lack of significant relationship between (SOFA) score in the two study groups can be the small sample size as well as the limited duration of the study.

It was also shown that the most dominant microorganisms responsible for infection in patient with (VAP) were gram-negative bacteria. This is in compliance with previous studies in which gram-negative bacteria were responsible for (MDR) infections in hospitalized patients (30, 31). This issue has created the necessity for using broad-spectrum antibiotics such as colistin that is now being increasingly used for treatment of these infections. 

Finally, it must be noted that vitamin D administration is correlated with the reduced mortality rate of patients with (VAP). This effect was independent of microorganism causing the infection and the therapeutic regiment.

In conclusion, our study revealed that (VAP) patients who suffer from vitamin D deficiency can benefit from a high-dose vitamin D injection. Its favorable impact was documented by reduced PCT levels and mortality rate in the intervention group compared to those of the placebo group. Further studies to clarify any relationship between mortality rate and vitamin D supplementation in (VAP) patients, with a larger sample size and for a longer follow-up period are recommended.
